# Mesh-Architected Structurally Flexible Pb(Zr_0.52_Ti_0.48_)O_3_ Framework Enables Highly Sensitive and Stretchable Piezoelectric Sensors

**DOI:** 10.1007/s40820-026-02148-1

**Published:** 2026-03-20

**Authors:** Li Zeng, Chenhui Jiang, Yuan Li, Hao Yin, Qichao Li, Hezhou Liu, Yiping Guo

**Affiliations:** State Key Laboratory of Metal Matrix Composites, School of Materials Science and Engineering, Shanghai Jiao Tong University, Shanghai, 200240 People’s Republic of China

**Keywords:** Piezoelectric sensor, Structurally flexible, High sensitivity, High stretchability, Mesh architecture

## Abstract

The booming demand for flexible electronics requires piezoelectric sensors that can simultaneously deliver high sensitivity and exceptional stretchability, which has become a significant challenge beyond the capabilities of conventional devices. Inspired by the hexagonal mesh structure, we adopted an architecture design to fabricate a continuous, structurally flexible ceramic skeleton, thereby developing a piezoelectric sensor integrating both high sensitivity and superior stretchability. The macroscopic 3D interconnected skeleton ensures efficient stress transfer for enhanced pressure sensitivity, while the hexagonal topology with hierarchical microfibers enables deformation-driven slippage under load to ensure robust stretchability. Consequently, the composite achieves remarkable stretchability (220% strain) and excellent mechanical stability (> 50 stretch-compression cycles, hysteresis ~ 8.13%). The fully flexible piezoelectric sensor maintains stable functionality even under 100% tensile strain and exhibits high sensitivity (39.57 mV kPa^−1^), collectively outperforming conventional piezoelectric sensors. Based on these unique advantages, we demonstrate the sensor’s capability in fine surface roughness discrimination and real-time monitoring of human stretching movements, highlighting its great potential for applications in robotic dexterous manipulation and wearable health monitoring systems.

**Supplementary Information:**

The online version contains supplementary material available at 10.1007/s40820-026-02148-1.

## Highlights

- A monolithic, structurally flexible, continuous Pb(Zr_0.52_Ti_0.48_)O_3_ (PZT) piezoelectric fiber was innovatively fabricated through network architecture design.
- The PZT framework well inherited the macro- and micro-structure of the mesh fabric, endowing the PZT-silicone composite material with high stretchability up to 220%.
- The developed strain sensor exhibited both high stretchability (100%) and high sensitivity (39.57 mV kPa^−1^), thus enabling its application in subtle surface roughness perception and large-strain human motion monitoring.

**Supplementary Information:**

The online version contains supplementary material available at 10.1007/s40820-026-02148-1.

## Graphical abstract

**Figure:**
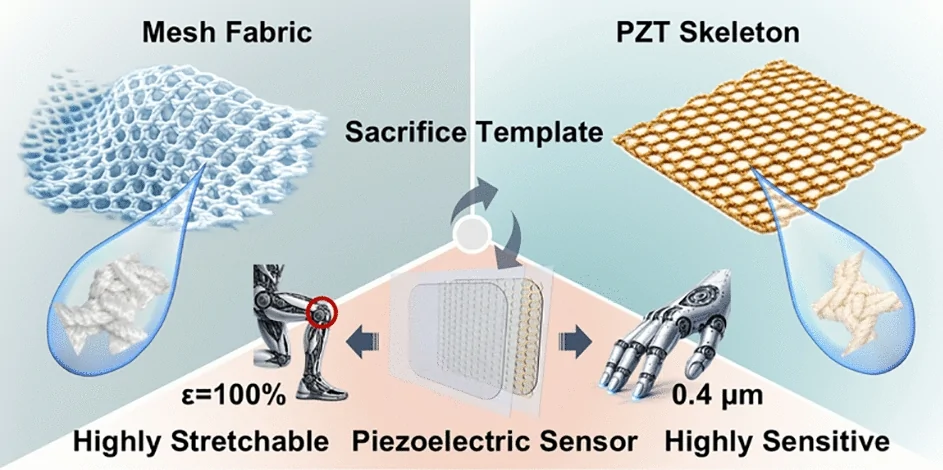

## Introduction

Flexible piezoelectric sensors are essential for converting mechanical signals into electrical signals and demonstrate considerable potential in the fields of health monitoring, human–machine interfaces, and soft robotics [1–5]. To operate effectively in dynamic or highly extensible environments, these sensors must conform to deformable surfaces (e.g., articulated joints) while simultaneously detecting subtle stimuli [6–8]. Consequently, this imposes stringent requirements for sensors to achieve both high stretchability and high sensitivity [9–11]. These requirements are fundamentally limited by an intrinsic materials trade-off. Strong piezoelectric responses arise from rigid inorganic ceramics with long-range ferroelectric ordering and efficient stress-transfer pathways, but their fracture strains are typically limited, whereas polymer-based materials capable of large deformation possess low polarization order and piezoelectric coefficients generally below 30 pC N^−1^ [12], resulting in insufficient signal output for high-sensitivity detection. There is an inherent trade-off between mechanical compliance and piezoelectric performance, which makes it highly challenging to achieve adaptive sensing in wearable systems operating at articulated joints and in soft robotic platforms [13, 14].

To achieve high-performance flexible piezoelectric sensors, the research community has extensively explored methods to enhance their stretchability and sensitivity [15–17]. However, efforts have largely proceeded along two distinct pathways. In the pursuit of high sensitivity, studies demonstrate that beyond selecting materials with high piezoelectric coefficients, meticulous microstructural design is paramount. Notably, constructing interconnected three-dimensional (3D) piezoelectric networks has proven to be an effective strategy, which can be synthesized via sacrificial templating [18–20]. Such architectures facilitate highly efficient stress transfer and distribution through their continuous skeletons under external loading [21–23]. This mechanism significantly enhances piezoelectric output and sensitivity. However, these inherently rigid 3D structures typically cannot withstand large tensile deformations. Even with elastic encapsulation, their continuous skeleton, geometrically not being a kinematic unit, remains difficult to stretch [24, 25]. In contrast, research focused on achieving high stretchability more frequently draws upon established experience from the flexible electronics field. Designing polymeric piezoelectric materials (e.g., PVDF) into serpentine meandering shapes or kirigami structures represents two representative approaches [26, 27]. For instance, Yan et al. patterned PVDF into a serpentine mesh layout, enabling the intrinsically difficult-to-stretch piezoelectric thin films to achieve the desired stretchability (tensile strain ~ 27%) [26]. The serpentine structure dissipates tensile stress through out-of-plane bending, while the kirigami structure exhibits lateral expansion upon stretching due to Poisson’s ratio effect. This synergistic interaction endows the integrated device with substantially enhanced stretchability. Regrettably, these patterning-oriented strategies frequently fail to achieve concurrent optimization of the device’s sensitivity. In certain instances, the introduction of non-functional elastomeric components or a reduction in active sensing layer density through these approaches results in suboptimal piezoelectric sensitivity [26, 28].

While interconnected 3D structures provide a clear micro-level mechanism for achieving high sensitivity through enhanced stress transfer efficiency, and serpentine or kirigami architectures offer a reliable macro-scale solution for enabling high stretchability, few studies have successfully synergistically integrated these advantageous mechanisms from distinct scales within a single system. To simultaneously achieve both high sensitivity and high stretchability in devices, it is necessary to transform rigid ceramic materials with high piezoelectric coefficients into structurally mobile units [29], demanding effective flexibilization of such materials.

This study achieves the transformation of Pb(Zr_0.52_Ti_0.48_)O_3_ (PZT), a material with a high piezoelectric coefficient, from rigidity to structural flexibility through mesh architecture design, successfully fabricating a piezoelectric sensor that concurrently exhibits high sensitivity and high stretchability. We employed ultrasonic spraying combined with a sacrificial template method to enable the PZT material to inherit the macro- and micro-morphology of a mesh fabric. Macroscopically, an interconnected 3D piezoelectric network was formed, which facilitated efficient stress transfer under load, converting localized forces into a global response, thereby contributing to high sensitivity. Its hexagonal mesh structure exhibited a kirigami-like effect; during stretching, transverse elongation accompanies longitudinal contraction, and multi-level fiber sliding within the fabric dissipates stresses, thereby contributing to high stretchability. The stretchability of this PZT composite reached 220%. Furthermore, the as-fabricated piezoelectric sensor achieved 100% stretchability, maintained mechanical stability after over 50 stretch-compression cycles, exhibited voltage output stability after 12,500 compression tests, and demonstrated a high sensitivity of 39.57 mV kPa^−1^. Owing to its high sensitivity, the sensor could detect human radial artery pulse waveforms and discriminate surface roughness as low as 0.4 μm. Due to its excellent stretchability, it can be utilized to monitor the rehabilitation progress of the human knee joint.

## Experimental Section

### Materials

The chemicals, 2-methoxyethanol, acetylacetone (AR), lead acetate trihydrate (AR, SCR), zirconium n-propoxide (70 wt%), and titanium butoxide (≥ 99%) were purchased from Aladdin. All chemicals were used without further purification. The mesh fabric was from a specialty textile fiber factory (thickness: 0.5 mm).

### Preparation of the PZT Solution

To compensate for lead volatilization during high-temperature sintering, an additional 10% of lead acetate was used. The synthesis route of a typical PZT sol with a concentration of 0.6 mol L^−1^ was as follows: in ethylene glycol methyl ether solvent, first added acetylacetone as a complexing agent (to slow down the hydrolysis rate of the Ti source), then sequentially added Pb, Zr, and Ti sources in accordance with the molar ratio. The mixture was stirred at 70 °C for 4 h until the solution turned into a clear bright yellow color, and then aged at room temperature for 48 h for subsequent use.

### Preparation of PZT Piezoelectric Sensor

The PZT skeleton was prepared based on the sacrificial template method. A mesh fabric template (15 cm × 15 cm × 0.5 mm) was coated with the PZT sol using an ultrasonic spray coater (YMUS-ZM200) operating at 80 kHz. Compressed air (0.5 MPa) was used as the carrier gas. The nozzle speed was 20 mm s^−1^, flow rate 10 μL s^−1^, and spray width 8 mm. After coating, the template was annealed at 1000 °C for 2 h to form the PZT skeleton (heating rate at 2 °C min^−1^). The PZT skeleton was then polarized by corona poling (80 °C, 13 kV, 70 min), cut into 1 cm × 1 cm pieces, and encapsulated with silicone rubber.

The PZT piezoelectric sensor adopted a simple sandwich structure. To avoid damage to the stretchability caused by the electrode layer, we used flexible conductive fabric as the electrode, which was directly covered on the upper and lower sides of the PZT. The entire structure was filled and infiltrated with silicone rubber, forming a fully flexible and stretchable piezoelectric sensor.

### Finite Element Simulation

Given the structural complexity of the actual PZT network, X-ray micro-computed tomography (micro-CT) was first employed to obtain three-dimensional images of the architecture. A three-dimensional fiber-bundle reconstruction algorithm integrating threshold segmentation and Gaussian smoothing was then applied to extract the macroscopic yarn structure. The extracted yarn network was meshed using iso2mesh and directly adopted as the PZT skeleton model, while the silicone rubber matrix and the PZT skeleton were assigned to different material domains via region labeling. The reconstructed mesh was imported into ABAQUS for mechanical analysis, and a tie constraint was imposed at the skeleton–matrix interface to prevent relative separation during loading. Boundary conditions were defined by fixing one end of the model and applying a prescribed displacement to the opposite end to induce tensile deformation, enabling the mechanical response to be obtained and analyzed. The details are shown in Note S1.

### Characterization and Measurements

We used Thermogravimetry–Differential Scanning Calorimetry (TG-DSC, Netzsch STA449 F3) to determine the mass change of the mesh fabric template during sintering at 1000 °C (testing atmosphere: Oxygen-Argon blend; heating rate: 10 °C min^−1^); the crystalline phase structure of the sintered PZT framework was tested by X-Ray Diffraction (XRD, Rigaku D/MAX 2400, Cu Kα radiation), its morphology was characterized by Scanning Electron Microscopy (SEM, NERCN-TC-006), and its ferroelectric response was characterized by Piezoresponse Force Microscopy (PFM) mode of an atomic force microscope (AFM, Bruker Multimode 8). The mechanical properties of the PZT-silicone rubber (PZT-SR) composite material and the PZT piezoelectric sensor were measured using a mechanical testing machine (ZQ-990) at a stretching and releasing speed of 60 mm/min. The reciprocating compression and tensile movements of the sensor were controlled by a linear motor, and the real-time output signals were recorded by a Keithley 6514 electrometer. The resistance change was measured by a Keithley 2420 multimeter.

## Results and Discussion

### Design of the Highly Sensitive and Stretchable PZT Piezoelectric Sensor

**Fig. 1:**
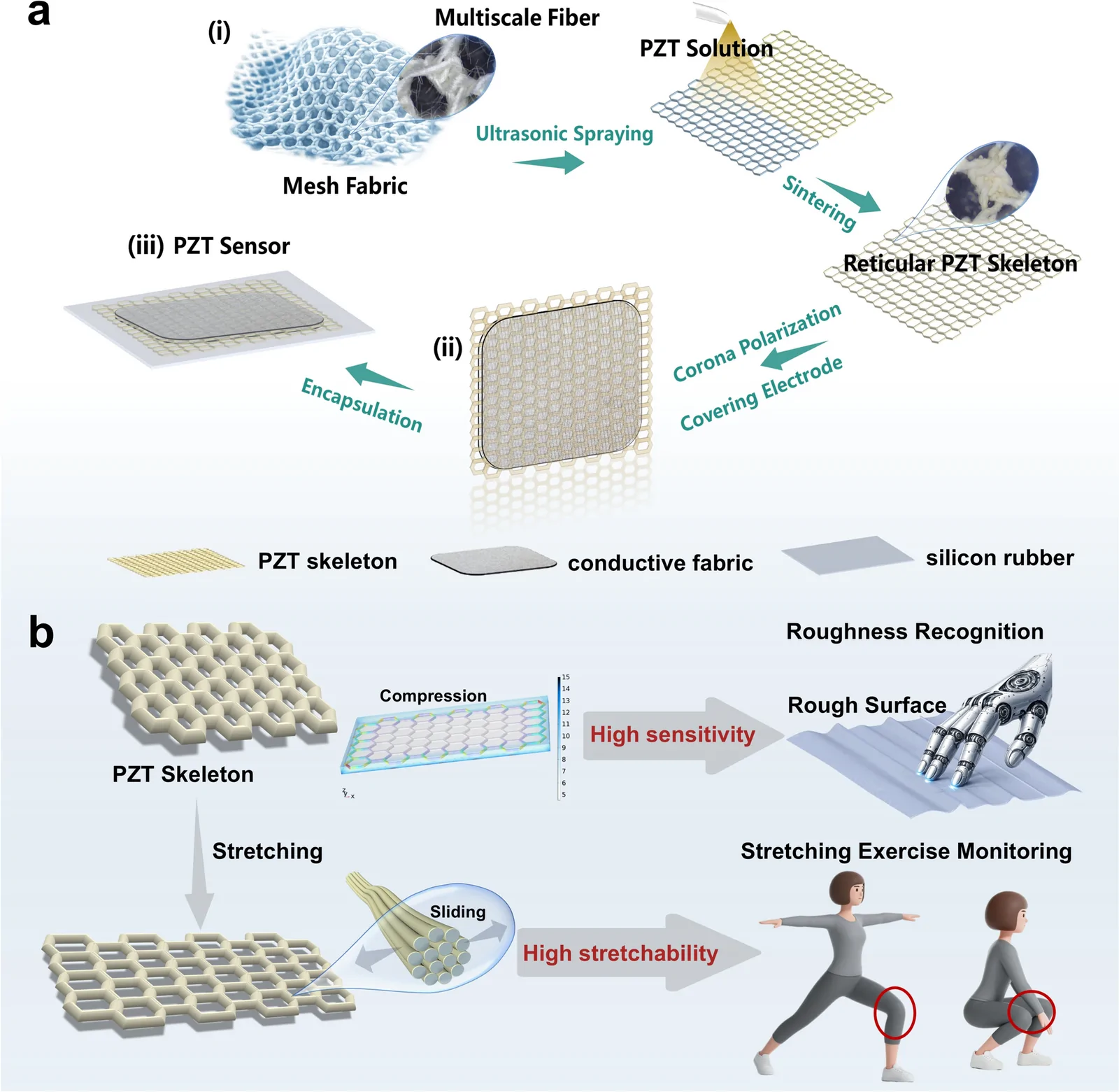
**a** Design of the PZT sensor. **(i)** The fabrication process transforms mesh fabric into the PZT skeleton. The mesh fabric possesses a macroscopic network structure and a microscopic hierarchical fiber architecture. This structure is replicated onto the PZT piezoelectric material using ultrasonic spraying combined with a sacrificial template method. **(ii)** Magnified views depict the sensing layer and electrode structure. **(iii)** A schematic of the overall PZT sensor structure employs a straightforward sandwich configuration. Fabric electrodes form the top and bottom layers, encapsulating a central PZT skeleton, with the entire assembly sealed in silicone. **b** Application scenarios

Hexagonal mesh fabric (Fig. 1a(i)) exhibits unique macro- and micro-scale morphologies and geometric structures. Macroscopically, while the fabric is porous, it features an interconnected 3D structure. This macro-scale interconnectivity facilitates stress transfer within the sensing material. When an external force is applied to a specific region of the sensing layer, the stress at that location is effectively transmitted to other areas. This promotes the overall mechano-electrical response of the material, contributing to the achievement of high sensitivity. Microscopically, the mesh fabric possesses a multi-level fiber architecture. Specifically, the knots are woven from multiple coarse fibers, and each coarse fiber is itself formed by twisting together multiple fine fibers. Under external tensile force, the multi-level fibers within the knots undergo slippage. This slippage dissipates energy, thereby preventing fracture caused by stress concentration during stretching. Additionally, due to the Poisson’s ratio effect, the hexagonal structure experiences geometric deformation under tension, which also facilitates the preparation of stretchable materials.

To replicate the macro and micro-structural characteristics of mesh fabric, we employed an ultrasonic spraying technique combined with a sacrificial template approach to fabricate a PZT ceramic framework. Through mesh-like structural design, this ceramic skeleton exhibited flexibility, enabling the subsequent fabrication of a highly sensitive and stretchable PZT piezoelectric sensor. Figure 1a(i) illustrates the fabrication process of the PZT framework. Ultrasonic spraying enabled large-area production. Following high-temperature sintering at 1000 °C, the resulting PZT framework retained the original grid structure of the mesh fabric, preserving the hierarchical fibers at the network junctions.

After corona poling, we employed a straightforward sandwich configuration to assemble the PZT sensor device (Fig. 1a(ii)). Conductive fabric served as the electrode material. The inherent softness and stretchability of the conductive fabric (Fig. S1) prevented any compromise to the overall stretchability of the device. Finally, the PZT piezoelectric layer and conductive fabric electrodes were co-encapsulated within a flexible silicone elastomer (Fig. 1a(iii)). The porous nature of both the PZT framework and the electrodes allowed the silicone to wet and infiltrate the interfaces, effectively preventing electrical shorting between the top and bottom electrodes. Owing to its high sensitivity, the PZT sensor can detect subtle surface roughness. Furthermore, the device’s excellent stretchability facilitated the monitoring of large-strain human motions (Fig. 1b).

### Synthesis and Characterization of the PZT Framework and Derived Composite

**Fig. 2:**
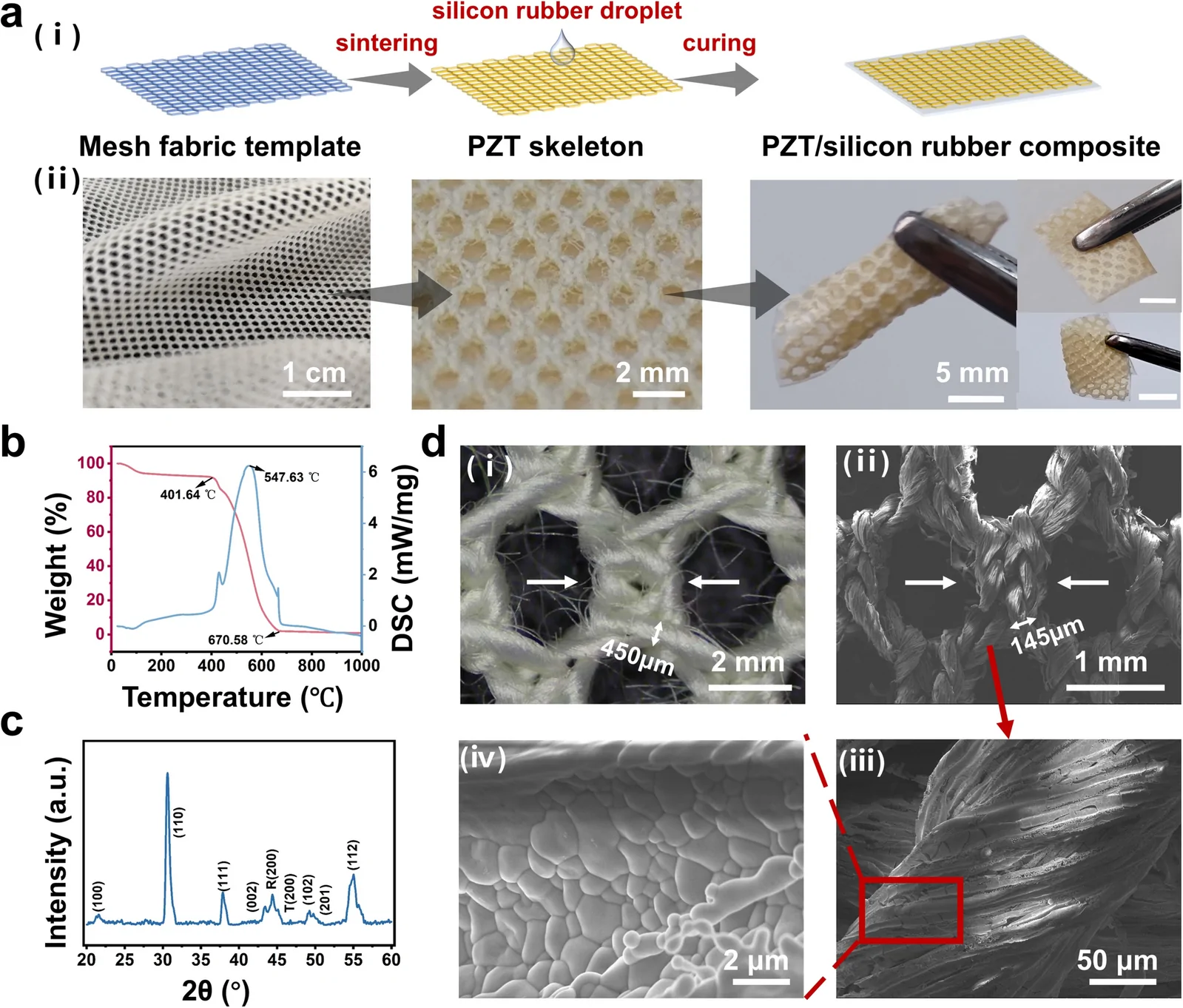
**a (i)** Fabrication process of the PZT-SR composite. **(ii)** Corresponding photograph of the fabrication process. **b** TG-DSC curve of the pure mesh template measured from room temperature (RT) to 1200 °C. **c** XRD pattern of the PZT skeleton after high-temperature sintering at 1000 °C. **d (i)** Optical micrograph of the mesh template. **(ii)–(iv)** SEM images of the PZT skeleton after high-temperature sintering, captured at progressively higher magnifications: **(ii)** network junction, **(iii)** individual coarse fiber, **(iv)** grains on a single coarse fiber

The PZT skeleton was fabricated using ultrasonic spraying combined with a sacrificial template method. As shown in Fig. 2a(i), the mesh fabric served as the template. A uniform PZT sol was ultrasonically sprayed onto this template. Subsequent annealing and high-temperature sintering completely oxidized and removed the mesh fabric template. Concurrently, the metallic networks within the PZT sol interconnected and underwent nucleation and crystallization, yielding a yellow ceramic skeleton [30]. To preserve structural integrity, the skeleton was infiltrated and encapsulated with silicone, resulting in the PZT-silicon rubber composite material (Fig. 2a(ii)).

To ensure the phase purity of the PZT skeleton obtained after sintering at 1000 °C, characterization was performed on both the pure mesh template and the skeleton for verification. Firstly, TG-DSC analysis of the mesh template was conducted from room temperature (RT) to 1200 °C (Fig. 2b). The relative weight of the mesh template decreased continuously throughout the sintering process, exhibiting a sharp decline at 401 °C and approaching zero near 670 °C. This indicates the complete oxidative removal of the template at this stage. The DSC curve revealed a significant endothermic peak at 547 °C, corresponding to the period of rapid mass loss. Our sintering program employed a maximum temperature of 1000 °C, ensuring the sacrificial template was fully removed while enabling the crystallization of the PZT sol at high temperature, as subsequently verified by XRD. As shown in Fig. 2c, the XRD pattern of the PZT skeleton exhibits the characteristic perovskite structure. Among these, the relative diffraction intensity of the (100) plane was the highest, which may indicate the presence of a preferred orientation. The splitting of the diffraction peak near 45° into three distinct peaks, and the differentiation of the (200) peak into R(200) and T(200) components collectively indicate the coexistence of rhombohedral and tetragonal phases [31]. This coexistence enhances the number of effective polarization directions, which is beneficial for polarization switching under minor external stress [32–34]. This characteristic is advantageous for achieving high sensitivity in subsequent piezoelectric sensors.

Optical microscopy and SEM were employed to verify the replication of the original mesh fabric structure by the PZT skeleton. As shown in Fig. 2d(i), the pristine mesh fabric exhibited a repeating pattern of mesh holes and intertwined fiber knots, with a hole width of approximately 2 mm and a fiber diameter of about 450 μm. The PZT skeleton sintered at 1000 °C (Fig. 2d(ii)) successfully replicated the mesh pores and complex knots of the original textile template, exhibiting only slight dimensional shrinkage, with a pore size of approximately 1 mm and an individual fiber diameter of about 145 μm. This faithful structural replication can be attributed to the excellent shape retention of the template and the densification behavior of PZT during high-temperature sintering. Local magnified images (Fig. 2d(iii)) show that the fibers at the knot regions retained the twisted morphology of the original template, while the grain size was approximately 1.09 μm (Fig. 2d(iv)). The incomplete densification between grains is believed to help mitigate brittle fracture under external loading (Fig. S2) [30, 35]. From a microstructural perspective, both the fracture morphology and internal architecture of the PZT skeleton contribute to its flexible deformation capability (Fig. S3). Fracture at the knot regions exhibits a pronounced concave morphology, resembling the dimple-like fracture features of ductile materials rather than the flat, brittle fracture surfaces typical of dense ceramics, indicating that local damage can be alleviated through energy dissipation and structural rearrangement during loading. Meanwhile, the skeleton interior displays a loose and porous structure, containing numerous non-densely contacted skeletal regions and partially or non-contacting grains. This architecture significantly reduces rigid constraints between grains, allowing stress to be dispersed through intergranular micro-sliding and progressive contact reconfiguration, thereby avoiding stress concentration. These multiscale structural features are critical for achieving flexibility and enhanced fracture resistance in the inherently brittle PZT skeleton (Fig. S4a, Video S1).

In addition, the density of the PZT ceramic skeleton was measured to be approximately 6.2 g cm^−3^ (Note S2), which was lower than the theoretical density of dense PZT ceramics. Nitrogen BET adsorption measurements (Fig. S4b) further revealed a high porosity and a significantly reduced effective ceramic volume fraction, providing structural evidence in support of the proposed flexible deformation mechanism based on incomplete grain contacts and inter-fiber sliding. The high porosity effectively reduces the number of load-bearing contact points and alleviates stress concentration, serving as a key factor enabling flexibility in the intrinsically brittle PZT mesh skeleton.

Overall, the PZT skeleton effectively inherits the structural features of the textile template at both macroscopic and microscopic levels, resulting in a structurally flexible PZT skeleton that provides a solid foundation for achieving high sensitivity, stretchability, and overall flexibility in subsequent piezoelectric sensor applications.

**Fig. 3:**
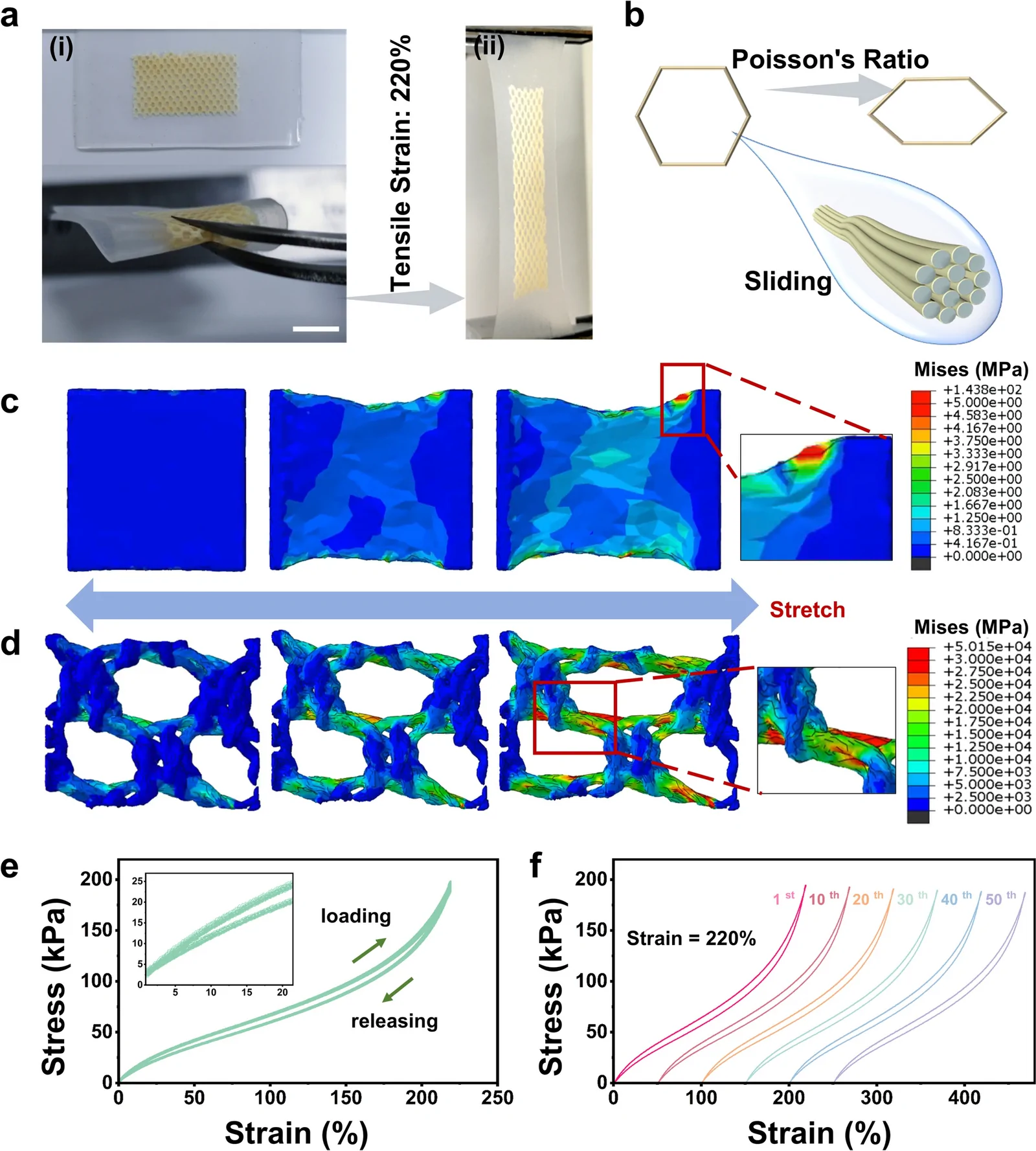
Mechanical characterization of the PZT-silicone elastomer 3–3 composite. **a** Schematic of tensile deformation, showing axial elongation with transverse contraction, **(i)** initial state before tensile testing (scale bar: 1 cm) and **(ii)** optical image after stretching. **b** Deformation mechanism under tension: macroscopic deformation of the hexagonal mesh induced by Poisson’s effect and microscopic deformation accommodated by sliding within the hierarchical fibrous structure. Finite element simulations of stress distribution under tensile strain, **c** stress distribution of the composite surface, and **d** stress distribution of the PZT skeleton from the initial to the tensile state. **e** Nearly overlapping stress–strain curves over 50 consecutive loading–unloading cycles at 220% strain, indicating excellent cyclic stability. **f** Tensile stress–strain curves recorded at the 1 st, 10th, 20th, 30th, 40th, and 50th cycles

The 3–3 composite formed by the PZT skeleton and silicone elastomer exhibits outstanding mechanical properties. Firstly, due to the excellent wettability and infiltration capacity between the silicone and the PZT skeleton, the composite achieved a remarkable tensile strain up to 220% (Fig. 3a(i) and (ii), Video S2), significantly exceeding existing stretchable piezoelectric sensors (Table S1). During stretching, the composite’s hexagonal mesh structure (Fig. 3b) deformed under the Poisson’s ratio effect, elongating transversely while contracting longitudinally. Compared to alternative stretchable architectures (e.g., serpentine and Kirigami), this mesh design offers distinct advantages in simplicity and ease of fabrication, eliminating the need for complex patterning processes. Furthermore, because the PZT skeleton inherited the hierarchical fibrous structure of the template, energy dissipation occurred under tensile loading via sliding between the infiltrated coarse fibers, thereby preventing stress concentration and fracture [36]. The stress distribution of the composite under tensile strain was further investigated through theoretical simulations (Fig. 3c, d). The simulation model was reconstructed from textile CT images (Note S1 and Fig. S5), as shown in Fig. S6, with the silicone rubber treated as a rectangular matrix encapsulating the PZT skeleton. Figure 3c illustrates the surface stress distribution of the composite during progressive stretching from the initial state, where the silicone rubber surface exhibited a largely uniform stress distribution, with stress concentration observed only in small edge regions. Figure 3d shows the stress distribution within the PZT skeleton. During stretching, multi-level fibers undergo gradual sliding. The peripheral fibers of each hexagonal unit display similar stress distribution characteristics, with stress mainly concentrated in fibers aligned with the tensile direction. Moreover, the stress difference between the horizontal and vertical directions is relatively small, and no large-area stress concentration is observed, thereby effectively suppressing tensile fracture. Based on this understanding, the composite was subjected to 50 consecutive stretching-compression cycles at 220% strain. As shown in Fig. 3e, the stress–strain curves for these cycles are nearly overlapping, indicating exceptional elastic recovery and demonstrating the material’s mechanical durability. A magnified view (Fig. 3f) reveals that the hysteresis loss, calculated using Eq. (1) [37]:

1 $$\begin{array}{*{20}c} {H = \frac{{A_{{\mathrm{L}}} - A_{{\mathrm{R}}} }}{{A_{{\mathrm{L}}} }} \times 100\% } \\ \end{array}$$

where H refers to the hysteresis ratio, *A*_L_ represents the area enclosed by the loading curve and the strain axis, and *A*_R_ represents the area enclosed by the releasing curve and the strain axis. According to Eq. (1), hysteresis loss was only 8.13% at the 50th cycle. This combination of high stretchability and low hysteresis ensures the reliability of this piezoelectric material under large tensile strains.

### Mechanical and Electrical Properties of the PZT Sensor

**Fig. 4:**
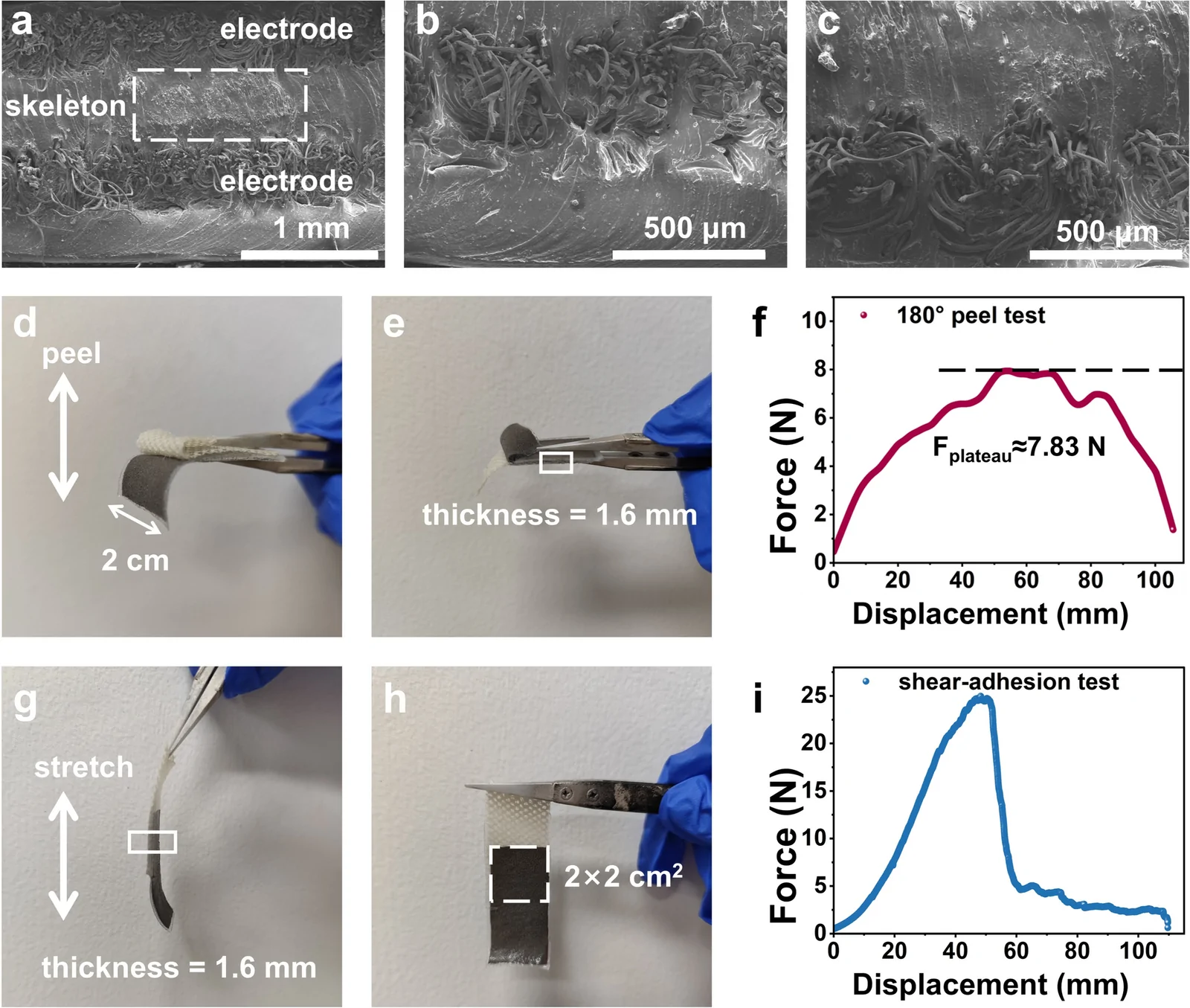
**a** Cross-sectional morphology of the sandwich-structured sensor. **b** Enlarged view of the interface between the bottom electrode and the PZT layer. **c** Enlarged view of the interface between the top electrode and the PZT layer. **d**-**f** Parameters and results of the 180° peel test specimens. **g**–**i** Shear strength test specimens and corresponding test results

The PZT sensor was prepared from the fabric-templated PZT framework, and optical images and dimensional details of the PZT sensor are provided in Fig. S7. The interfacial bonding characteristics between the silicone and the PZT as well as the conductive textile are shown in Fig. S8. Benefiting from the porous structures of the conductive textile and the PZT skeleton, together with the excellent wettability of silicone rubber on the textile surface, the silicone rubber can readily flow and infiltrate into both the conductive textile and the PZT, thereby forming a robust mechanical interlocking structure. The cross-sectional morphology of the sensor was shown in Fig. 4a–c, where the device was entirely encapsulated by silicone rubber, resulting in an integrated structure with strong bonding among the functional layers. The interfacial bonding strength was further quantitatively evaluated by 180° peel and shear tests. As shown in Fig. 4d–f, the maximum peel force reached 8.2 N, and a stable force plateau was observed at a displacement of approximately 53–70 mm, with an average plateau force of 7.83 N. With a specimen width of 2 cm, the apparent interfacial toughness was calculated using the relation [38]:

2 $$\begin{array}{*{20}c} {{\text{interfacial toughness}} = \frac{{2F_{{{\mathrm{plateau}}}} }}{W}} \\ \end{array}$$

yielding a value of 783 J m^−2^. In addition, the shear strength test shown in Fig. 4g–i indicated a maximum shear force of 24.8 N, demonstrating the excellent interfacial bonding and mechanical stability of the sensor [39].

**Fig. 5:**
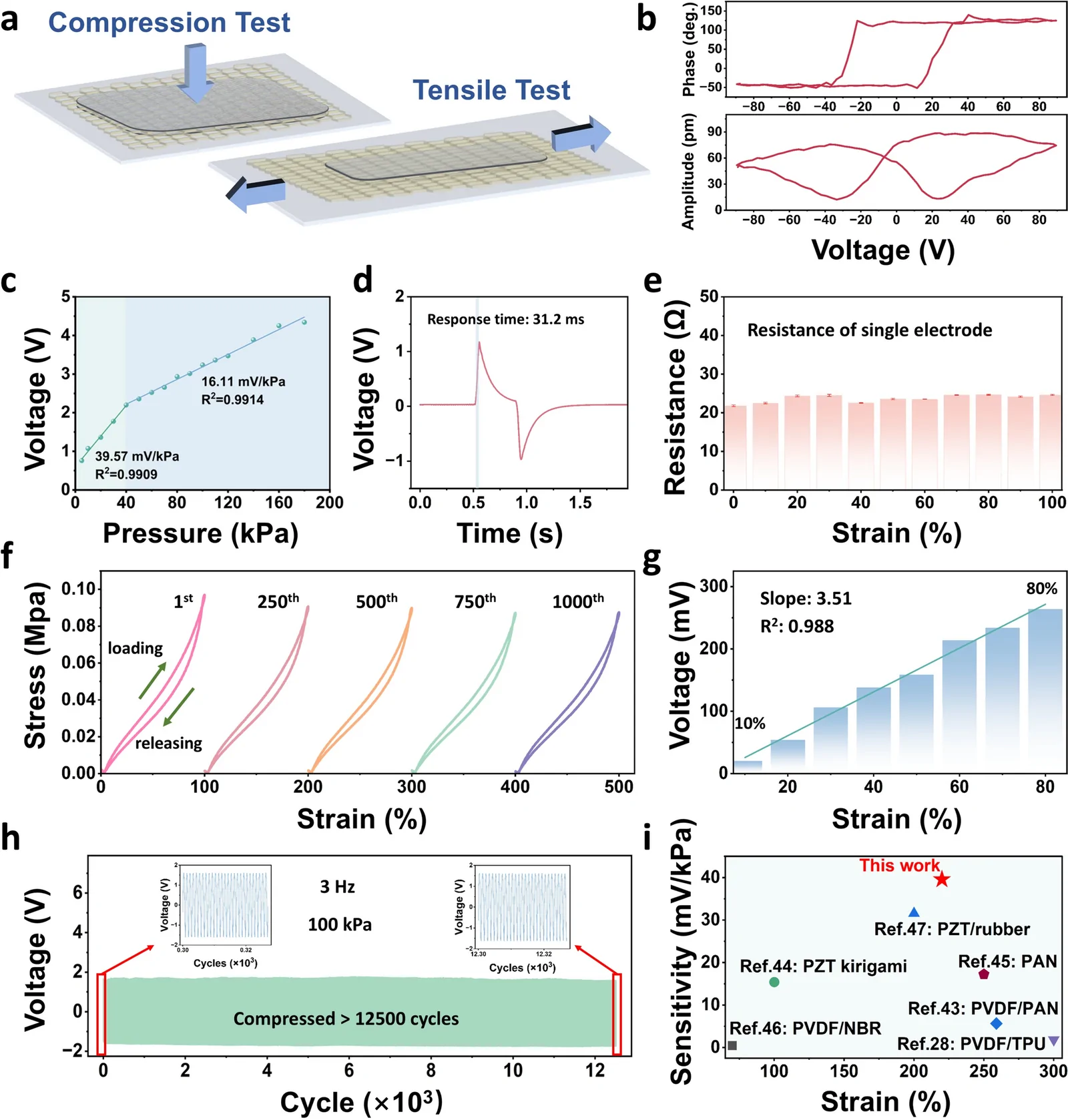
**a** Sensing performance test of the PZT sensor in compression mode and tension mode, respectively. **b** Piezoresponse force microscopy (PFM) phase and amplitude images of the piezoceramic skeleton. **c** Linear relationship between pressure and output voltage amplitude (tested at 3 Hz on a 1 cm^2^ area). **d** Response time of PZT sensor under compressive force testing. **e** Variation in electrode resistance during the stretching of the PZT sensor from its initial state to 100% strain. **f** Tensile stress–strain curves of PZT sensor at 100% strain after the 1st, 250th, 500th, 750th, and 1000th cycles. **g** Voltage amplitude and corresponding linearity analysis for tensile strains ranging from 0 to 80%. **h** Endurance testing under compression (3 Hz, 100 kPa) mode. **i** Comparison of pressure sensitivity and stretch ratio between this work and other studies [28, 43–47]

Subsequently, it is necessary to conduct a sensing performance test on this PZT sensor (Fig. 5a). Before this, the switchable polarization of the PZT material after high-temperature sintering was confirmed via PFM, validating its suitability for piezoelectric sensors. In Fig. 5b, the characteristic 180° phase switching and the well-defined symmetric “butterfly-shaped” loop of the amplitude curve are indicative of ferroelectric behavior [40]. The amplitude-voltage response curve reveals a coercive voltage of approximately ± 20 V, signifying that polarization switching can be achieved at relatively low voltages. A maximum amplitude of 80 pm was attained, demonstrating strong piezoelectric activity and making the material suitable for the design of high-sensitivity sensors.

The sensing performance of the device was evaluated under both compression and tension modes to achieve a high-sensitivity, stretchable integrated design. In the compression mode, a PLA loading block with a base area of 1 cm^2^ was employed to apply a periodic normal force to the PZT sensor surface at a frequency of 3 Hz. Under out-of-plane compressive loading, deformation occurred along the thickness direction of the PZT sensor. The voltage signal output was measured under applied normal loads ranging from 5 to 180 kPa (corresponding to 0.5–18 N). As shown in Fig. 5c, the voltage-load response curve exhibited a two-stage sensitivity characteristic: the device demonstrated a high sensitivity of 39.57 mV kPa^−1^ in the low-pressure range of 5–40 kPa, which subsequently decreased to 16.11 mV kPa^−1^ in the range of 40–180 kPa. This segmented sensitivity difference has been reported in other studies [16, 41]. Since the sensitivity in the d_33_ mode of the sensor is related to deformation along the thickness direction, the PZT sensor readily deforms under low-pressure loads [42]. As the applied pressure increased from 5 to 40 kPa (0.5–4 N), the deformation along the thickness direction of the PZT sensor increased significantly. Under high pressure loads, the PZT sensor gradually approaches its deformation limit in the thickness direction; consequently, even with larger applied loads, the deformation is less pronounced compared to that in the low-load region, resulting in the observed decrease in sensitivity. The corresponding voltage-load waveform signals are provided in Fig. S9. A segment of the waveform was extracted to calculate the compressive response time of the PZT sensor, which is approximately 31.2 ms, as shown in Fig. 5d. The durability of the device under compression mode is shown in Fig. 5h. After being tested for over 12,500 cycles at 3 Hz under a 100 kPa normal force, the voltage output remained stable, indicating that the PZT sensor is suitable for long-term use.

For operation in tensile mode, the sensor’s functionality necessitates stretchable conductive pathways within the electrodes [27]. To achieve this, electrical leads were connected to one side of the electrode. The PZT sensor was mounted on a mechanical testing machine and subjected to tensile strain tests ranging incrementally from 0 to 100% strain. Concurrently, the resistance of the single-sided electrode was monitored using a Keithley 2420 source meter. As presented in Fig. 5e, the initial resistance of the textile electrode was approximately 20 Ω. Upon sequential stretching to 100% strain, the resistance exhibited a slight increase but remained within the range of 20–25 Ω. This indicates that the sensor maintains effective electrical connectivity throughout the entire 100% strain range. The stress–strain curves obtained from 1000 consecutive stretching-compression cycles at 100% strain are shown in Figs. 5f and S10. The near-overlapping curves demonstrated excellent cyclic stability. Furthermore, at 100% strain, the PZT sensor exhibited enhanced tensile strength compared to PZT-SR composites, attributed to the incorporation of the textile electrode. Moreover, the maximum force during stretching was only 1.6 N (Fig. S10a), which was far below the maximum interfacial peel or shear force, demonstrating that stretching the device to 100% strain is mechanically safe. Subsequently, a linear motor was employed to apply periodic tensile and release cycles to the sensor to evaluate its voltage output under varying tensile strains. Instantaneous voltage signals were recorded using a Keithley 6514 electrometer. As shown in Fig. 5g, the voltage output increased from 0 to 263.93 mV as the strain rose from 0 to 80%. The high linearity (R^2^ = 0.988) observed over this large strain range can be attributed to the uniform stress distribution within the PZT sensing material. To assess the viability of the PZT sensor for high-strain applications, its durability was evaluated under cyclic loading controlled by the linear motor at 3 Hz and 70% strain (Fig. S11). After approximately 6700 cycles, a voltage output attenuation of 15.57% was observed. This degradation is primarily attributed to relaxation occurring at the interface between the silicone matrix and the clamping fixtures after repeated deformation. In summary, the design successfully integrates high sensitivity with high stretchability. This integrated performance demonstrates superior characteristics compared to similar sensor configurations (Fig. 5i, Tables S1 and S2).

### Surface Roughness Identification and Human Stretching Motion Monitoring

PZT sensor offers high pressure sensitivity and rapid response, enabling accurate detection of microscopic surface roughness, which is critical for tactile sensing in robotic manipulators. Its superior flexibility, high stretchability, and stable mechanical/electrical properties ensure seamless conformal contact with curved surfaces. These capabilities support monitoring of extensive body movements, including knee joint rehabilitation exercises. Before practical application, the environmental stability of the sensor was verified. As shown in Fig. S12, benefiting from the inertness of the silicone rubber encapsulation layer, the sensor exhibited stable performance under varying humidity levels, ultraviolet irradiation, and operating temperatures.

**Fig. 6:**
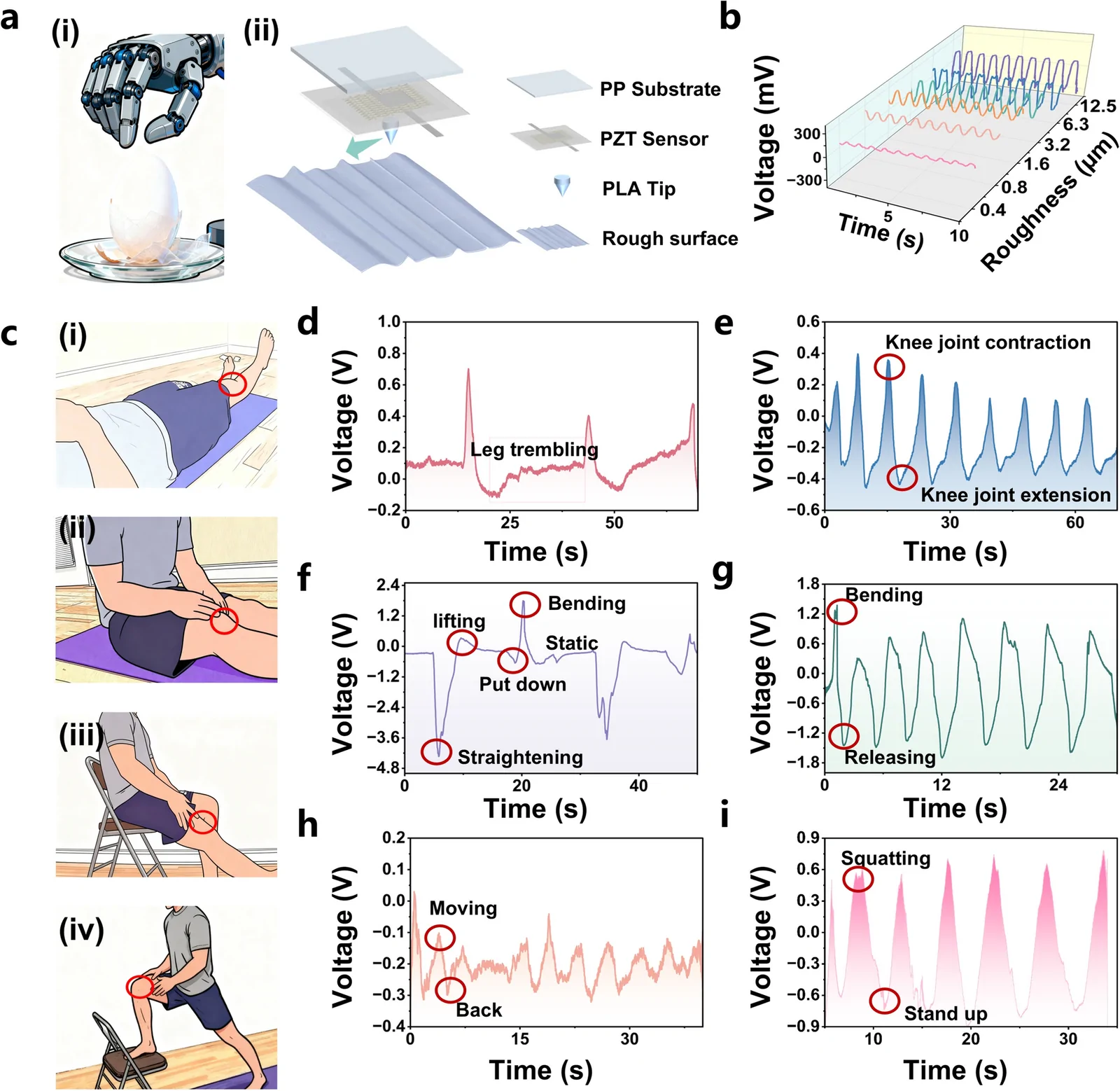
**a (i)** Surgical robot training for eggshell removal (simulating delicate human tissue) requires precise pressure control to prevent irreversible damage. **(ii)** Schematic of surface roughness testing. **b** Roughness measurement results. **c (i)–(iv)** Knee rehabilitation exercises. The position marked with a red circle is where the PZT sensor should be attached. **d–i** Corresponding voltage signal outputs and interpretations during rehabilitation motions

Tactile sensors are vital for advancing dexterous robotic hands. Precise pressure detection at the micron scale is particularly crucial for surgical robots (Fig. 6a(i)) performing delicate operations [48]. Accordingly, our PZT sensor was employed for micron-level roughness detection. To validate its high sensitivity, we first tested the sensor on the human radial artery (Fig. S13). The clear triphasic pulse waveform demonstrated its micro-force detection capability. We then applied it to roughness measurement using the setup in Fig. 6a(ii): A PLA tip, the PZT sensor, and the PP plate were bonded together and mounted on a linear motor. This assembly functioned like a macroscopic atomic force microscopy probe. When the tip encountered surface protrusions during translation, vertical forces deformed the PZT sensor, generating electrical signals via the piezoelectric effect. We conducted tests using standard roughness blocks (0.4–12.5 μm range, Fig. S14). As shown in Fig. 6b, the voltage amplitude increased with surface roughness. This confirms our sensor can detect forces from 0.4 μm protrusions, showing significant potential for enhancing precision in surgical robotics.

The PZT sensor demonstrates exceptional performance in knee joint rehabilitation monitoring. The sensor’s high stretchability enables seamless contact with the skin during knee movements, allowing real-time tracking of biomechanical parameters. Compared to conventional devices, its thin, conformal design eliminates motion restrictions, offering a revolutionary solution for dynamic post-surgery monitoring. We securely attached the sensor to the lateral knee using medical PU tape (Fig. S15). Its superior stretchability permits continuous surface stretching/recovery during knee flexion–extension cycles, whereas low-stretchability devices (< 5%) would experience structural failure under similar conditions.

Six rehabilitation exercises were sequentially performed. First, the straight leg raise (Fig. 6c(i)): Subjects lay supine with the left leg extended and the right leg lifted straight upward, held briefly, then lowered. This exercise targets muscle strength recovery and joint protection. The sensor output (Fig. 6d) showed three voltage peaks corresponding to lifting motions. During static holds, voltage returned rapidly to baseline, with minor fluctuations indicating muscle tremors. Progressively decreasing peak amplitudes across the three lifts demonstrated muscle fatigue, signaling the need for rest. Second, knee straightening (Fig. 6c(ii)): Seated subjects repeatedly extended a slightly bent knee with manual assistance to enhance muscle coordination and lower-limb function. Figure 6e shows voltage peaks during knee flexion and inverse waveforms during extension, generated by sensor deformation. Third, sitting knee straightening: Seated subjects lifted one leg from vertical to horizontal without manual assistance, holding briefly before returning. This restores terminal knee extension while reducing joint load. Corresponding signals appear in Fig. 6f. The fourth exercise, active knee bend (Fig. 6g), required subjects to lie prone while supporting their upper body with hands, repeatedly bending knees toward the back and returning. Consistent, short intervals between voltage peaks indicated effective knee recovery. Decreased signal amplitude or prolonged peak intervals suggested muscle fatigue, warranting rest periods. The fifth exercise, step-up lunges (Fig. 6c(iv)), integrates strength, balance, and functional training. Using a stool, subjects maintained one leg extended for support while performing small-range flexion–extension motions in a lunge position with the other foot. Sensor outputs (Fig. 6h) revealed unstable voltage waveforms, indicating compromised balance control. Minor signal fluctuations further demonstrated residual tremors, suggesting ongoing muscle strength recovery is required. The last exercise, 1/4 squats (Fig. 6i), targets explosive strength development and rehabilitation during joint sensitivity phases. Voltage waveforms exhibited consistent amplitudes, demonstrating good knee stability and motor control in subjects. In summary, the PZT sensor successfully captures real-time voltage signals during all six rehabilitation exercises. Analysis of signal characteristics objectively quantifies key recovery metrics, including muscle fatigue, neuromuscular control, and joint stability. Compared with conventional devices, its superior conformal contact, sensitivity, and real-time feedback provide objective clinical data for optimizing treatment protocols and preventing re-injury. This technology offers an innovative solution with significant potential for advancement in rehabilitation medicine.

## Conclusion

This study utilized commercially purchased mesh fabric to structurally design high-piezoelectric-coefficient, hard PZT material via ultrasonic spraying combined with a sacrificial template method. This approach enabled the replication of the fabric’s macro and micro-structures, successfully fabricating a structurally flexible, continuous piezoelectric skeleton. Consequently, a flexible PZT piezoelectric sensor integrating both high sensitivity and high stretchability was developed. The intrinsic flexibility of the PZT skeleton arises from incomplete grain contact after sintering at 1000 °C, which hinders crack propagation during bending. The macroscopic 3D interconnected structure of the mesh enables holistic deformation under minor external forces, achieving a pressure sensitivity of 39 mV kPa^−1^. During tensile stress, hexagonal mesh units elongate along the loading direction due to Poisson’s ratio effects. Theoretical analysis confirms uniform stress distribution during stretching, yielding 220% stretchability in PZT-SR composites and 100% in the PZT sensor. The material and sensor exhibited mechanical stability during 50 cycles of 220% strain and 1000 cycles of 100% strain in the tensile recovery test. The sensor demonstrates practical utility in detecting micron-scale roughness (0.4 μm) and monitoring knee rehabilitation exercises, indicating promising applications for dexterous robotics and joint health assessment.

## Supplementary Information

Below is the link to the electronic supplementary material.

Supplementary file1 (MP4 3169 kb)

Supplementary file2 (MP4 4961 kb)

Supplementary file3 (DOCX 17882 kb)
