# Supplementary material for: Mesh-Architected Structurally Flexible Pb(Zr0.52Ti0.48)O3 Framework Enables Highly Sensitive and Stretchable Piezoelectric Sensors
Source: Nanomicro Lett. 2026 Mar 20;18:295. doi: 10.1007/s40820-026-02148-1 (PMC13004786; doi:10.1007/s40820-026-02148-1)
Supplement: Supplementary file 3 — Supplementary file3 (DOCX 17882 kb) [file 40820_2026_2148_MOESM3_ESM.docx]

Supporting Information for

**Mesh-Architected Structurally Flexible Pb(Zr_0.52_Ti_0.48_)O_3_ Framework Enables Highly Sensitive and Stretchable Piezoelectric Sensors**

Li Zeng^1^, Chenhui Jiang^1^, Yuan Li^1^, Hao Yin^1^, Qichao Li^1^*, Hezhou Liu^1^, and Yiping Guo^1^*

^1^ State Key Laboratory of Metal Matrix Composites, School of Materials Science and Engineering, Shanghai Jiao Tong University, Shanghai 200240, P. R. China

* Corresponding authors. E-mail: [liqichao@sjtu.edu.cn](mailto:liqichao@sjtu.edu.cn) (Qichao Li); [ypguo@sjtu.edu.cn](mailto:ypguo@sjtu.edu.cn) (Yiping Guo)

**Note S1 Finite element simulation**

The actual PZT network structure is relatively complex. To reconstruct its realistic geometry as accurately as possible, X-ray micro-computed tomography (micro-CT) was first employed to obtain three-dimensional images of the structure (**Fig.** 5a-c). A three-dimensional fiber-bundle reconstruction algorithm combining Gaussian smoothing and threshold segmentation was then applied to extract the macroscopic yarn structure from the voxelized microscopic fiber data.

As shown in **Fig.** 5d-f, during the model extraction process, the CT data were first segmented, and a binary mask of the fiber regions was obtained via threshold segmentation. Gaussian low-pass filtering was subsequently applied to the mask to generate a continuous density field, thereby enhancing the connectivity between adjacent fibers. The density field was then binarized using an appropriate threshold to extract the macroscopic yarn structure, from which the geometric and topological features of the networked fabric were obtained

For finite element simulations (**Fig.** 6), the extracted yarn structure was reconstructed into a computational mesh using iso2mesh with an appropriate mesh size. As the PZT skeleton inherited the macro- and microstructural characteristics of the networked fabric, the reconstructed yarn structure was directly adopted as the PZT skeleton model. The silicone rubber matrix and the PZT skeleton were then assigned to different material areas through region labeling. The parameters used are as follows.

The reconstructed mesh was imported into ABAQUS for mechanical analysis. In the simulations, the interfacial coupling between the skeleton and the silicone rubber matrix was not explicitly considered; instead, a tie constraint was applied to ensure that no relative separation occurred between the two phases during loading. Finally, one end of the model was fixed while a prescribed displacement was applied to the opposite end to impose tensile deformation, enabling the mechanical response to be obtained and analyzed.

**Note S2 Ceramic density measurement**

The ceramic density was measured using the Archimedes’ displacement method. The mass of the PZT skeleton measured in air (dry weight) was denoted as m **g**. The apparent mass of the PZT skeleton measured in water using a spring balance was denoted as M **g**. According to the force balance of the PZT skeleton in water,

$$\begin{aligned} mg=F+Mg\#\left( S1 \right) \end{aligned}$$

$$\begin{aligned} F=\rho Vg\#\left( S2 \right) \end{aligned}$$

where F is the Archimedean buoyant force, g is the gravitational acceleration, ρ is the density of water (1 g/cm³), and V is the volume of displaced water. Based on these relations, the displaced water volume can be determined, and the density of the PZT skeleton is calculated as：

$$\begin{aligned} \rho=\frac{m}{V}\#\left( S3 \right) \end{aligned}$$

In the experiment, the dry weight of the PZT skeleton was measured to be 0.108 g. The skeleton was then suspended on a spring balance in the water tank of a densitometer, yielding a wet weight of 0.091 g. Using the Archimedes’ displacement method, the calculated ceramic density of the PZT skeleton was 6.2 g/cm^3^.

**Supplementary Tables**

**Table S1** Comparison of this work with other studies on piezoelectric sensors

| Material | Max tensile strain (%) | Sensitivity (mV/kPa) | Stress detection range | Strain detection range | Response time | Durability cycles | References |
| --- | --- | --- | --- | --- | --- | --- | --- |
| BCZT ceramic array /PDMS/Ecoflex | 153 | 997.5 | 2 N-16 N | 0-60% | 24 ms | 5,000 | [S1] |
| BTO-PVDF-PU | ~40 | / | / | 0-40% | / | / | [S2] |
| PZT kirigami | ~100 | 15.4 | 30 kPa to 1.1 MPa | / | / | / | [S3] |
| PZT- rubber | ~200 | ~31 | / | / | / | / | [S4] |
| PVDF-PDMS | ~110 | / | / | 0-8% | / | 600 | [S5] |
| PVDF/PAN | 258.9 | 5.65 | 0-100 kPa | / | 15 ms | 10,000 | [S6] |
| Serpentine PVDF | 27.5 | / | / | 0-27.5% | / | / | [S7] |
| PVDF/TPU | ~300 | 1.83 | 0-70 N | / | 85 ms | >1,000 | [S8] |
| PAN | ~250 | 17.24 | 0.4-20 N | / | / | 10,000 | [S9] |
| PVDF-NBR | ~70 | 0.43 | 0-2 MPa | 0-70% | / | / | [S10] |
| **This work** | **220** | **39.57** | **0.5 N-18 N** | **0-80%** | **31.2 ms** | **>12,500** |  |

**Table S2** Comparison of this work with other studies on stretchable piezoresistive sensors

|  | Response time | strain detection range | stress detection range | Material elongation | Sensor elongation | References |
| --- | --- | --- | --- | --- | --- | --- |
| **This work** | **31.2 ms** | **0-80%** | **5 kPa-180kPa** | **220%** | **100%** |  |
| CuNW-rGO/PDMS | 135 ms | 0-65% | / | / | 70% | [S11] |
| Ti3C2MXene/PDMS | / | 0-70% | / | ~81% | / | [S12] |
| Carbon nanotubes /MXene | 158 ms | 0-105% | / | 203% | 105% | [S13] |
| Cellulose/MXene hydrogel | ~100 ms | 0-68.7% | / | ~144.4% | / | [S14] |
| Venetian fabric/WPU/CNTs | 32 ms | / | 0-45 kPa | 25% | / | [S15] |
| MWCNTs/bicomponent nonwovens | 100 ms | / | 0–120 kPa | ~40% | / | [S16] |
| PU/PPy NFs | 120 ms | 0-70% | 0–20kPa | 70% | 70% | [S17] |
| rGO/GO film | 72 ms | / | 0–40 kPa | / | / | [S18] |

**Supplementary Figures**


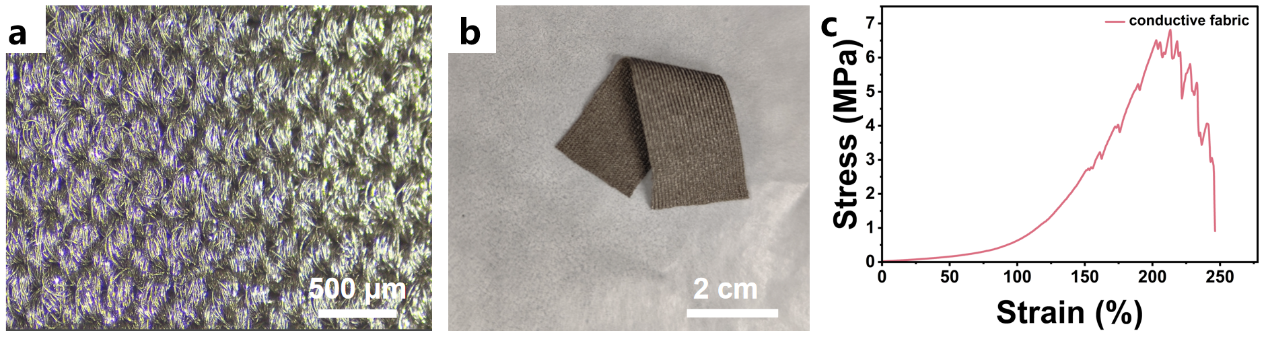


**Fig. 1** **a** Optical microscopy image, **b** photograph and **c** stress-strain curve of the conductive textile. The textile is commercially purchased, which is essentially fabricated by depositing a copper-nickel coating onto the surface of an elastic textile substrate via techniques such as magnetron sputtering. It has a thickness of approximately 0.2 mm

**Fig. 2** The individual fiber at the node of the PZT skeleton **a** and its magnified view **b** demonstrate incomplete contact between adjacent grains; **c**-**f** Non-densely contacted grains at different length scales.


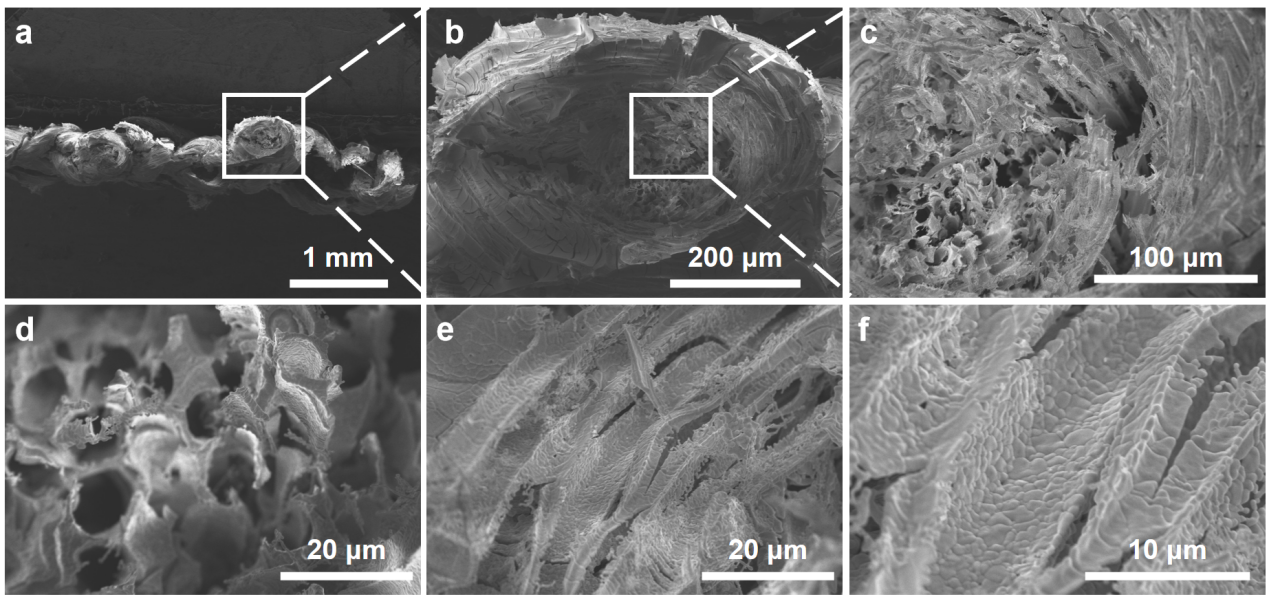


**Fig.** **3** **a** Fracture surface of the PZT skeleton; **b** Enlarged view of the interfacial knot region at the fracture surface; **c** Porous and non-densely contacted structure at the interface; **d** Through-pore morphology within the interfacial region; **e** Multilayered structure at the outer edge of the interface; **f** Enlarged view of the multilayered structure, showing non-dense grain contacts


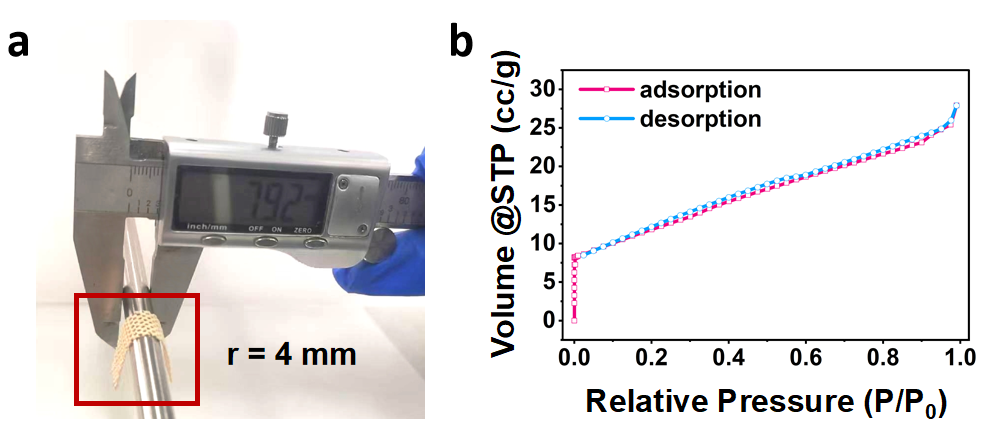


**Fig.** **4** **a** Bending curvature radius test of the PZT skeleton. **b** Nitrogen adsorption-desorption isotherm of the PZT skeleton measured by the BET method

**Fig.** **5** Representative images selected from 1,000 CT slice images. **a** Front view; **b**-**c** Side views. The brighter regions represent the textile structure. **d**-**f** Workflow for extracting the macroscopic yarn structure model from the original CT images

**Fig.** **6** Simulation model obtained from CT image processing


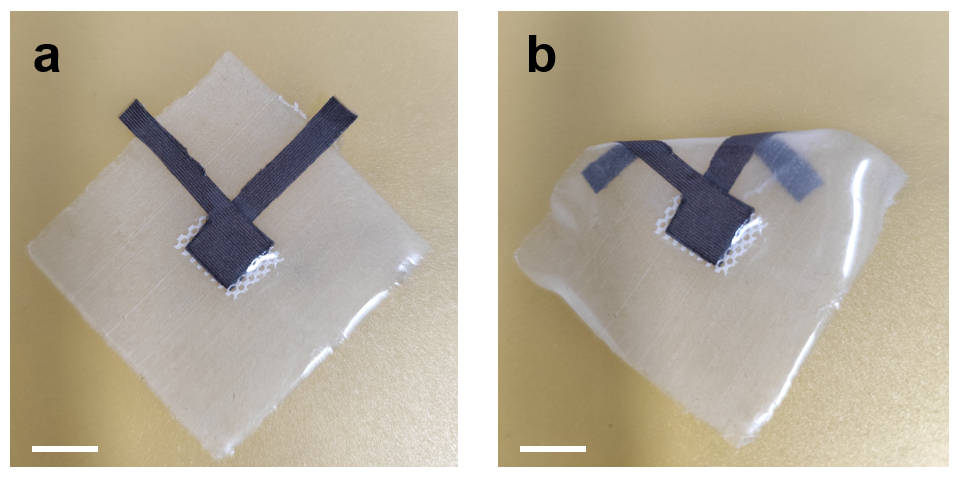


**Fig.** **7** Optical photographs of the PZT sensor. **a** Flat-laid state; **b** Bent state. Scale bar: 1 cm


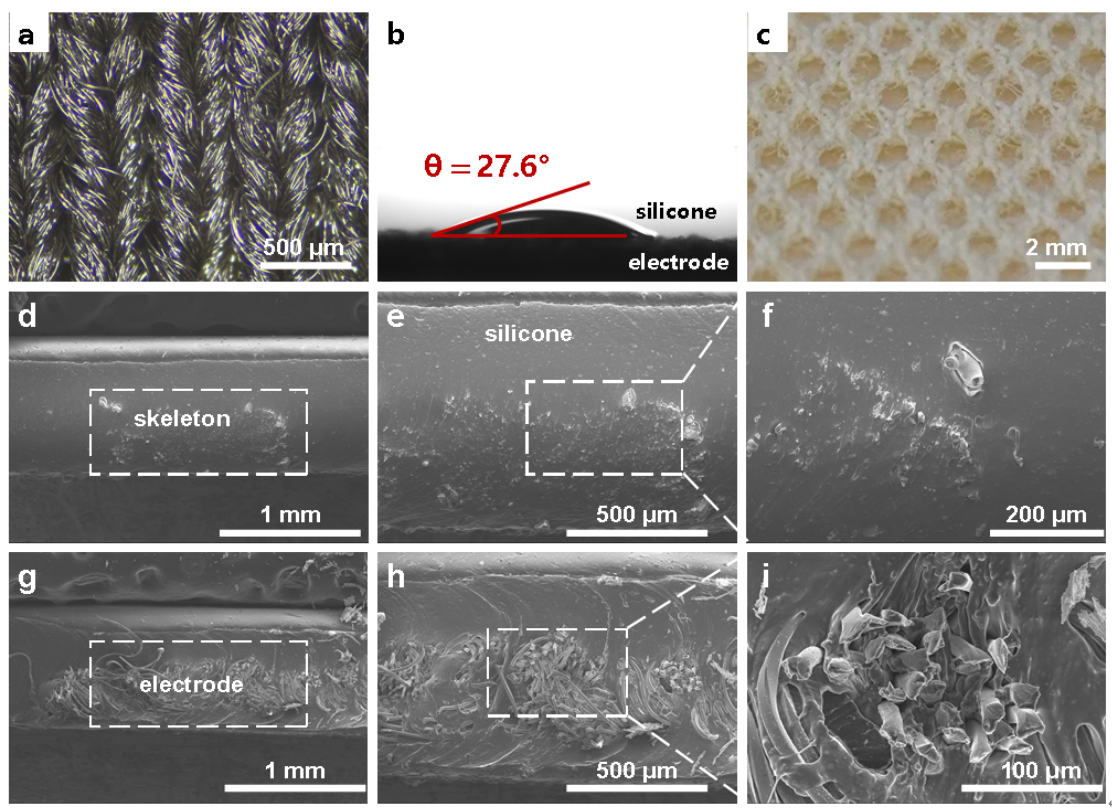


**Fig.** **8** **a** Optical morphology of the electrode; **b** Contact angle measurement of silicone rubber on the electrode; **c** Optical morphology of the PZT; **d**-**f** Cross-sectional morphologies of the silicone/PZT composite, showing that the PZT is fully infiltrated and encapsulated by the silicone rubber; **g**-(i) Cross-sectional morphologies of the silicone-electrode interface


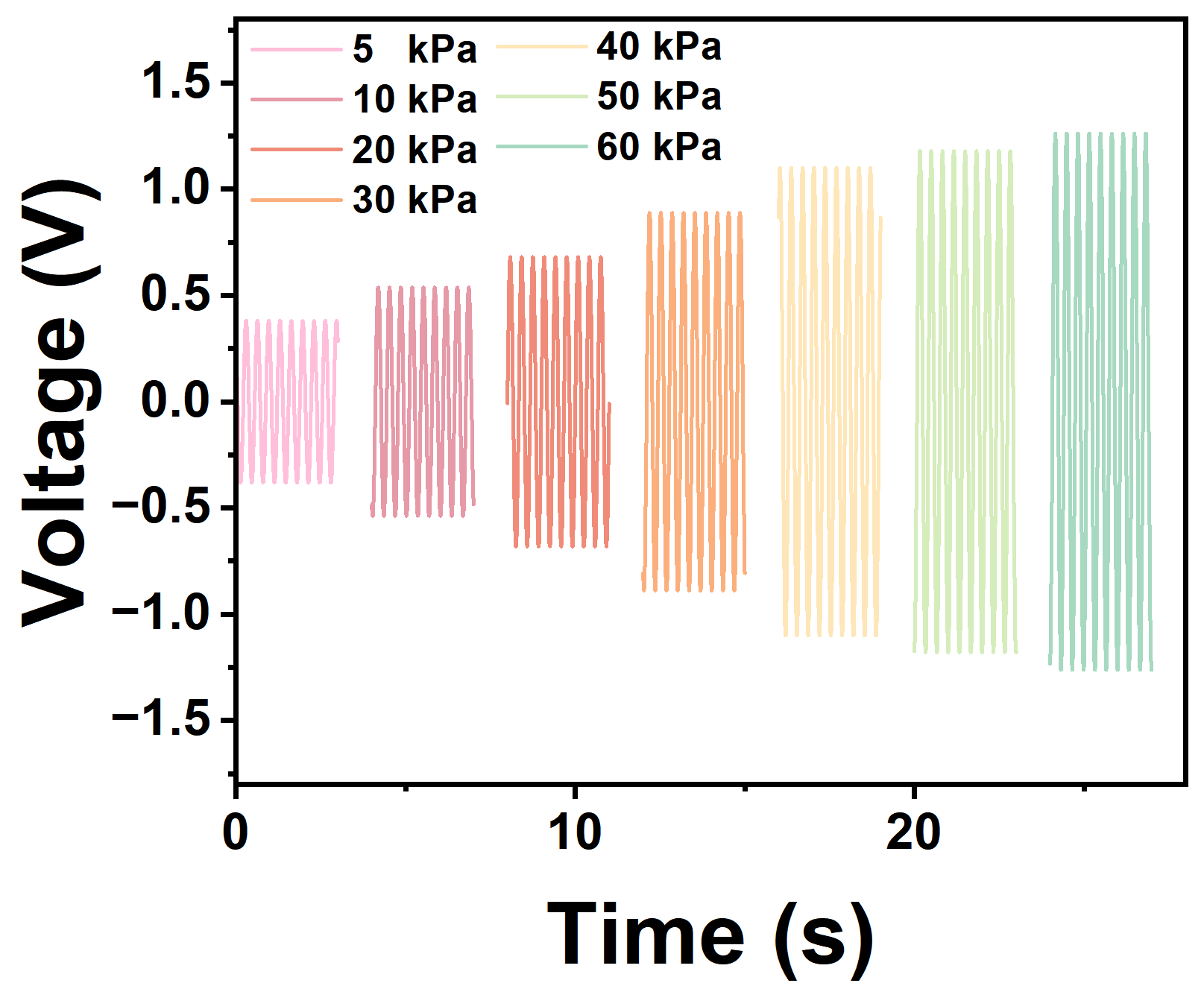


**Fig.** **9** The corresponding voltage-load waveform signals from 5 kPa to 60 kPa


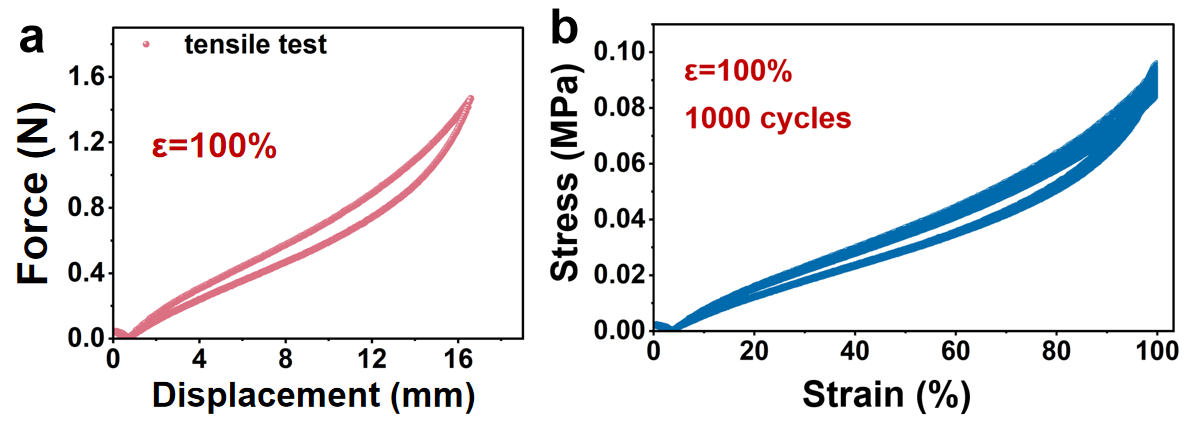


**Fig.** **10** **a** Force-displacement curve of the sensor during tensile testing; **b** 1000 tensile-releasing cycles of the sensor at 100% tensile strain


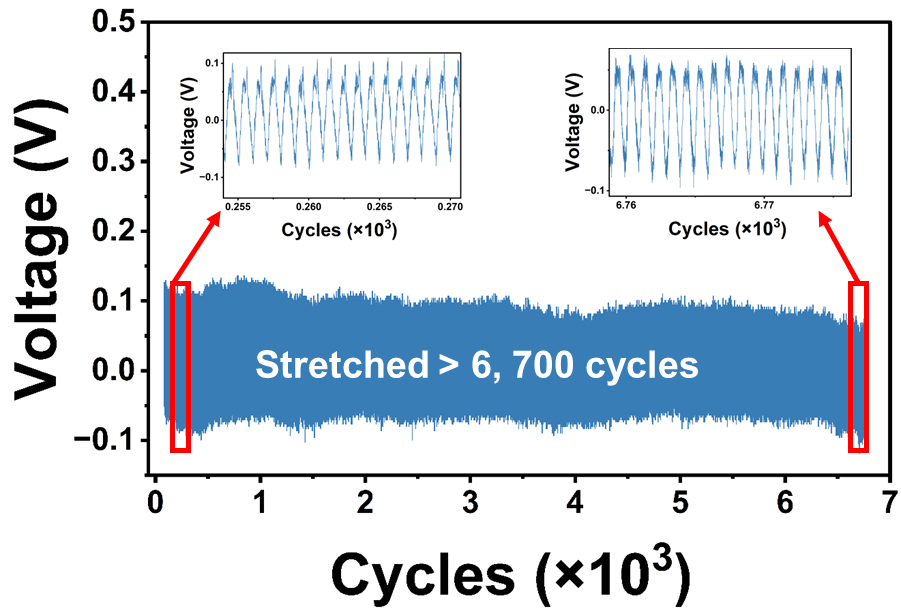


**Fig.** **11** Durability test under tensile mode over 6700 cycles at a 70% tensile strain and at a frequency of 3 Hz


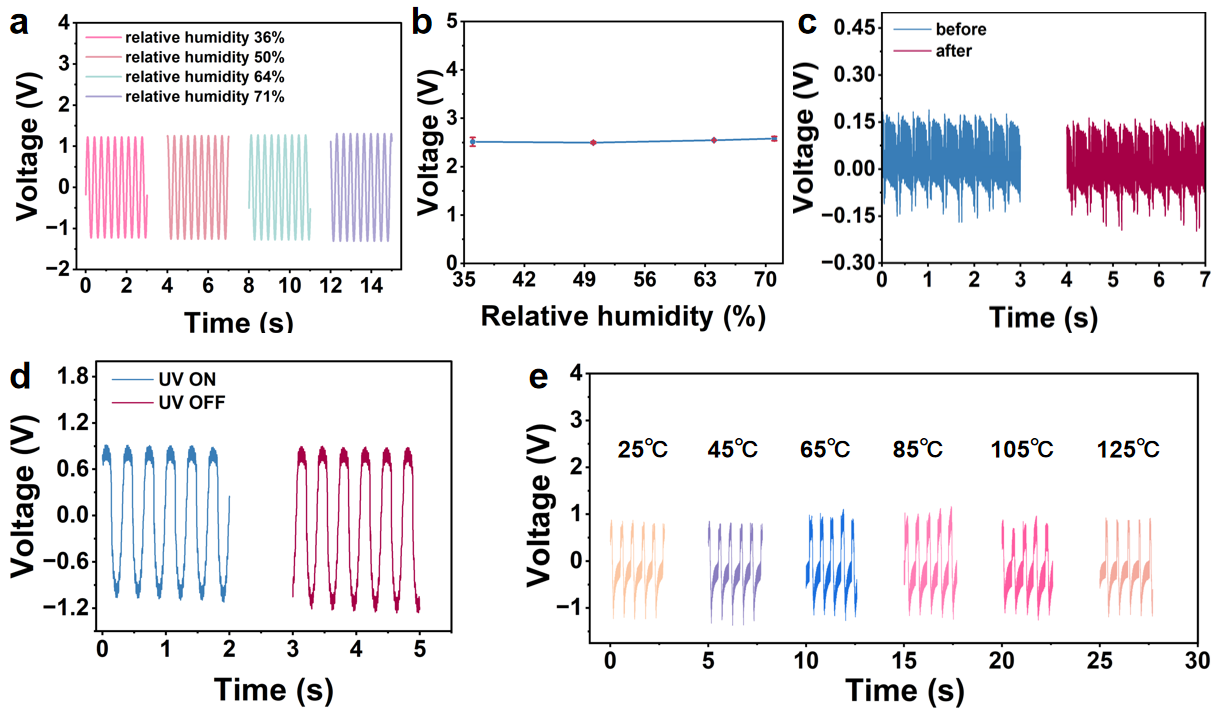


**Fig.** **12** **a**-**b** Voltage output waveforms of the sensor under different humidity conditions; **c** Voltage output waveforms of the sensor at 70% tensile strain before and after repeated bending when attached to the knee; **d** Voltage output of the sensor before and after ultraviolet irradiation (UV 254 nm, 60 min); **e** Voltage output of the sensor at different temperatures


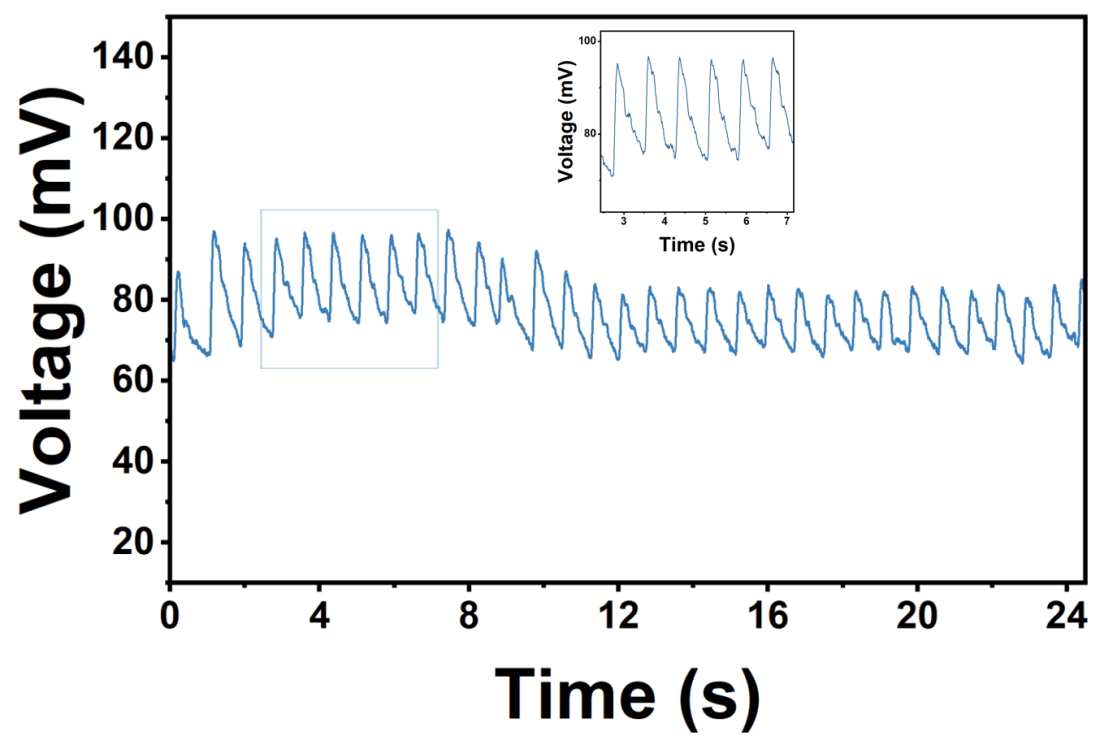


**Fig.** **13** Pulse waveform measured at the radial artery of the human wrist via affixed PZT sensor


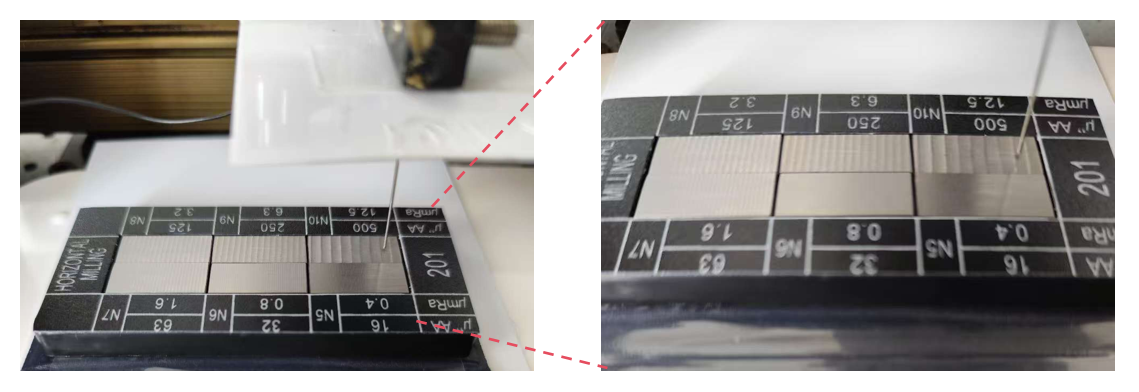


**Fig.** **14** Test Scenarios of Roughness Standard Blocks


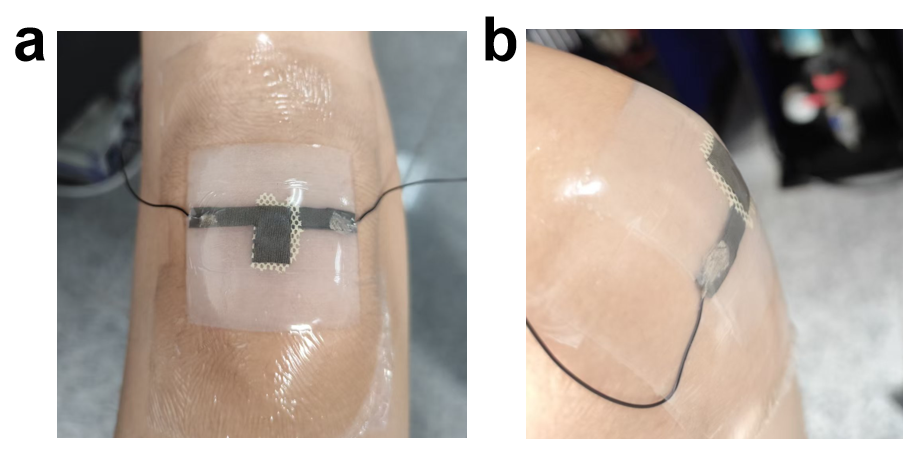


**Fig.** **15** **a** PZT sensors are affixed to the lateral aspect of the human knee joint using medical-grade polyurethane adhesive tape; **b** Tensile strain is generated in the PZT-PS composites during knee joint flexion

## Captions for Videos S1 and S2

**Videos S1** Demonstration of the Intrinsic Flexibility of the PZT skeleton by bending experiments.

**Videos S2** Demonstration of the high stretchability of the PZT-SR composite.

**Supplementary References**

1. Q. Xu, Y. Tao, Z. Wang, H. Zeng, J. Yang et al., Highly flexible, high-performance, and stretchable piezoelectric sensor based on a hierarchical droplet-shaped ceramics with enhanced damage tolerance. Adv. Mater. **36**(18), 2311624 (2024). <https://doi.org/10.1002/adma.202311624>
2. S. Siddiqui, H.B. Lee, D.-I. Kim, L.T. Duy, A. Hanif et al., An omnidirectionally stretchable piezoelectric nanogenerator based on hybrid nanofibers and carbon electrodes for multimodal straining and human kinematics energy harvesting. Adv. Energy Mater. **9**(11), 1900093 (2019). <https://doi.org/10.1002/aenm.201900093>
3. Y. Hong, B. Wang, W. Lin, L. Jin, S. Liu et al., Highly anisotropic and flexible piezoceramic kirigami for preventing joint disorders. Sci. Adv. **7**(11), eabf0795 (2021). <https://doi.org/10.1126/sciadv.abf0795>
4. X. Chou, J. Zhu, S. Qian, X. Niu, J. Qian et al., All-in-one filler-elastomer-based high-performance stretchable piezoelectric nanogenerator for kinetic energy harvesting and self-powered motion monitoring. Nano Energy **53**, 550–558 (2018). <https://doi.org/10.1016/j.nanoen.2018.09.006>
5. X. Yang, M. Zhang, P. Niu, W. Guo, C. Sun et al., Knee function assessment of anterior cruciate ligament injury with a Kirigami buckling-resistant stretchable sensor. SmartMat **5**(5), e1271 (2024). <https://doi.org/10.1002/smm2.1271>
6. Y. Guan, L. Tu, K. Ren, X. Kang, Y. Tian et al., Soft, super-elastic, all-polymer piezoelectric elastomer for artificial electronic skin. ACS Appl. Mater. Interfaces **15**(1), 1736–1747 (2023). <https://doi.org/10.1021/acsami.2c19654>
7. Z. Yan, T. Pan, D. Wang, J. Li, L. Jin et al., Stretchable micromotion sensor with enhanced sensitivity using serpentine layout. ACS Appl. Mater. Interfaces **11**(13), 12261–12271 (2019). <https://doi.org/10.1021/acsami.8b22613>
8. C. Wei, H. Zhou, B. Zheng, H. Zheng, Q. Shu et al., Fully flexible and mechanically robust tactile sensors containing core–shell structured fibrous piezoelectric mat as sensitive layer. Chem. Eng. J. **476**, 146654 (2023). <https://doi.org/10.1016/j.cej.2023.146654>
9. R. Fu, L. Tu, Y. Guan, Z. Wang, C. Deng et al., Intrinsically piezoelectric elastomer based on crosslinked polyacrylonitrile for soft electronics. Nano Energy **103**, 107784 (2022). <https://doi.org/10.1016/j.nanoen.2022.107784>
10. R. Tu, H.A. Sodano, Highly stretchable printed poly(vinylidene fluoride) sensors through the formation of a continuous elastomer phase. ACS Appl. Mater. Interfaces **15**(18), 22320–22331 (2023). <https://doi.org/10.1021/acsami.3c01168>
11. L. Cheng, D. Ruan, Y. He, J. Yang, W. Qian et al., A highly stretchable and sensitive strain sensor for lip-reading extraction and speech recognition. J. Mater. Chem. C **11**(25), 8413–8422 (2023). <https://doi.org/10.1039/D3TC01136D>
12. K. Zhang, J. Sun, J. Song, C. Gao, Z. Wang et al., Self-healing Ti_3_C_2_ MXene/PDMS supramolecular elastomers based on small biomolecules modification for wearable sensors. ACS Appl. Mater. Interfaces **12**(40), 45306–45314 (2020). <https://doi.org/10.1021/acsami.0c13653>
13. B. Xu, F. Ye, R. Chen, X. Luo, G. Chang et al., A wide sensing range and high sensitivity flexible strain sensor based on carbon nanotubes and MXene. Ceram. Int. **48**(7), 10220–10226 (2022). <https://doi.org/10.1016/j.ceramint.2021.12.235>
14. Y. Bai, S. Bi, W. Wang, N. Ding, Y. Lu et al., Biocompatible, stretchable, and compressible cellulose/MXene hydrogel for strain sensor and electromagnetic interference shielding. Soft Mater. **20**(4), 444–454 (2022). <https://doi.org/10.1080/1539445x.2022.2081580>
15. Q. Liu, Y. Zhang, X. Sun, C. Liang, Y. Han et al., All textile-based robust pressure sensors for smart garments. Chem. Eng. J. **454**, 140302 (2023). <https://doi.org/10.1016/j.cej.2022.140302>
16. G. Tian, Y. Shi, J. Deng, W. Yu, L. Yang et al., Low-cost, scalable fabrication of all-fabric piezoresistive sensors *via* binder-free, *in-situ* welding of carbon nanotubes on bicomponent nonwovens. Adv. Fiber Mater. **6**(1), 120–132 (2024). <https://doi.org/10.1007/s42765-023-00331-2>
17. X. Ren, Q. Tian, X. Zhu, H.-Y. Mi, X. Jing et al., Multi-scale closure piezoresistive sensor with high sensitivity derived from polyurethane foam and polypyrrole nanofibers. Chem. Eng. J. **474**, 145926 (2023). <https://doi.org/10.1016/j.cej.2023.145926>
18. M. Li, T. Wang, C. Han, H. Yang, Y. Huang et al., A novel piezoresistive sensor with rectification properties. Mater. Des. **239**, 112782 (2024). <https://doi.org/10.1016/j.matdes.2024.112782>
